# Supplementary material for: How Climate Warming Influences the Phenology of Grapholita molesta (Busck, 1916) (Lepidoptera: Tortricidae) in China: Insight from Long-Term Historical Data
Source: Insects. 2024 Jun 25;15(7):474. doi: 10.3390/insects15070474 (PMC11276667; doi:10.3390/insects15070474)
Supplement: Supplementary file 1 [file insects-15-00474-s001.zip › Table S2.pdf]

Table S2. Phenological records of *G. molesta* in different regions in China

**Region**	**Province**	**Valid record number of each province**
Central China	Henan	8
	Hubei	5
Eastern China	Anhui	25
	Fujian	3
	Jiangsu	42
	Jiangxi	6
	Shandong	60
	Shanghai	23
	Zhejiang	11
Northeastern China	Beijing	32
	Hebei	86
	Shanxi	80
	Tianjin	10
Northwestern China	Gansu	60
	Ningxia	16
	Shaanxi	24
	Xinjiang	54
Northern China	Heilongjiang	12
	Jilin	4
	Liaoning	30
	Inner Mongolia	7
Southwestern China	Guizhou	3
	Sichuan	9
	Yunnan	13
	Chongqing	3
Total		626
